# Watchful waiting in the treatment of the small renal mass

**DOI:** 10.4103/0970-1591.57923

**Published:** 2009

**Authors:** K. Clint Cary, Chandru P. Sundaram

**Affiliations:** Department of Urology, Indiana University School of Medicine, Indianapolis, IN, USA

**Keywords:** Active surveillance, kidney neoplasms, needle biopsy, natural history, small renal tumor

## Abstract

**Objectives:**

To evaluate the role and feasibility of observation with regard to the small renal mass.

**Methods:**

We performed a literature search of MEDLINE, reviewing the world literature relevant to the natural history, role of percutaneous biopsy and surveillance of the small renal mass.

**Results:**

The average yearly growth rate of most small renal masses ranges from 0.1 to 0.70 cm/yr with obvious exceptions. Clinical predictors of growth such as radiographic size at presentation, age, gender and tumor characteristics are not reliable. Approximately 1% develops metastatic disease while under surveillance. Contemporary series of percutaneous biopsy of small renal masses report sensitivity for malignancy to be 90%-98%. However, false-negative results can occur. For the majority of patients, the gold standard remains surgical extirpation.

**Conclusions:**

Watchful waiting is an acceptable option for management of small renal masses in the surgically unfit and elderly population. More information regarding the natural history and metastatic potential of small renal masses is needed. Percutaneous needle biopsy can be successful in detecting malignancy in selected patients with small renal masses. The role of needle biopsy for the small renal mass continues to evolve

## INTRODUCTION

The incidence of renal cell carcinoma (RCC) has been increasing worldwide for the past two decades. Incidental detection of small renal masses (SRMs) in the 1970s was as low as 7%-13%. This percentage has significantly increased to 48%-66% in the current literature largely because of the widespread use of computed tomography (CT).[1–4] The management of these masses in an individual patient is not always well defined. A growing body of literature supports active surveillance of the appropriately selected SRM in the elderly patient.[5] Recent reports have attempted to clarify issues such as the natural history, accuracy of biopsy, tumor growth rate, etc. of SRMs. Our objective in this review article is to provide current insights to help guide clinical decision making with regard to the possibility of observing the SRM.

## NATURAL HISTORY

The potentially aggressive nature of RCC and its resistance to systemic therapy is widely recognized. For this reason, the natural history of the SRM has not been fully investigated because most have been managed in an extirpative fashion.[6] Table 1 outlines the characteristics of recent major series in the literature with regard to active surveillance of the SRM. These studies used different types of imaging modalities during their follow-up period. Serial radiographic data on tumor growth rate alone probably will not give clinicians enough information to accurately predict the behavior of all SRMs. Further experience will be needed in order to define size thresholds for treatment. As seen in Table 1, most reports demonstrate the slow growth rates of SRMs. During an observation period, 26%-33% of incidental masses demonstrated zero net growth.[13] However, it must be remembered that this does not equal benign pathology. Several authors describe SRMs with no radiographic growth to be malignant on final surgical pathology.[7–9] In fact, Kunkle and colleagues demonstrated a striking finding where 83% of the masses demonstrating no growth were eventually pathologically proven to be RCC. Individual tumor biology is difficult to predict as evidenced here. More research involving tumor biology will provide more useful insight into more appropriate management. The authors advise that tumor growth rate alone will not be a reliable method to predict behavior of all SRMs. They advocate novel investigations are needed in cytogenetics, immunohistochemistry, etc. to provide more insight in predicting behavior.

**Table 1:** Select summary of the natural history of small renal masses

	No. masses	Initial tumor size (cm)	Growth rate (cm/yr)	% Proven malignant	Median length of follow-up (months)	Progression to metastatic disease (%)
Volpe *et al.* (2004)	32	2.48	0.10	89	28	0
Youssif *et al.* (2007)	44	2.2	0.24	75	41	5.7
Abouassaly *et al.* (2008)	110	2.5	0.26	60	24	0
Crispen *et al.* (2008)	124	2.0	0.21	90	26	1.4
Wehle *et al.* (2004)	29	1.83	0.12	80	32	0
Kassouf *et al.* (2004)	26	3.3	0.49	100	24	0
Kouba *et al.* (2007)	46	2.92	0.70	87	36	0

There are rare reports of progression to metastatic disease. Youssif *et al.* had two patients who went on to develop metastatic disease (5.7%). However, one patient was lost to follow-up and represented with spinal cord compression due to metastasis and the other was offered surgical resection after rapid tumor growth but deferred treatment for a total of 26 months then developed metastatic disease shortly after nephrectomy. Therefore, these two cases could have potentially been prevented if they had followed the observation protocol. The growth rate for these two tumors was approximately 0.9 cm/yr. The authors concluded that the majority of SRMs demonstrate slow growth and surveillance must be restricted to carefully selected patients as observation carries a significant risk. Crispen *et al.* also reported one patient who progressed during the observation period. The patient presented at 84 years of age with a 2-cm tumor and a rapid growth rate of 1.3 cm/yr. At a 54-month follow-up multiple pulmonary lesions were noted.

Several limited series are found throughout the literature regarding the natural history of SRMs.[10,11,14,15] Most are small and retrospective, and thus it is difficult to derive concrete principles for use in clinical management. A recent meta-analysis performed by Chawla *et al.* evaluated 286 renal lesions. Mean initial lesion size at presentation was 2.6 cm. Lesions were followed up for a mean of 34 months and the growth rate was found to be similar to other reports at 0.28 cm/yr. Of 286 lesions, 131 (46%) had pathological data available. Of those, 92% were found to be malignant. The range of malignancy in each series was between 80% and 100%. They found that the growth rate of pathologically confirmed RCC was significantly greater than lesions that were continued to be observed. The authors suggested a possibility for this is that a higher percentage of lesions under continued observation were benign. They found 3 patients (1%) who developed metastatic disease while under observation at 54, 111 and 132 months of follow-up. They could not find any correlation between lesion size at presentation and growth rate, which is consistent with other reports in the literature. Initial tumor size and growth rates of pathologically confirmed oncocytomas (9 of 76) and RCC variants (67 of 76) masses were compared. Tumor size at presentation for oncocytomas and RCC variants were 2 and 2.21 cm, respectively. No statistical difference was noted in growth rate between the two groups. Ultimately, they concluded that observation of the SRM is a calculated risk between patient and physician. In an appropriately selected elderly patient with significant comorbidities, this can be a viable option. Currently, we do not have a noninvasive method to differentiate benign vs. malignant tumors. They suggest like many studies that more research in translational and clinical outcomes are needed.

Some recently published preliminary data correlate radiologic findings with the histological subtype of renal tumor. A recent retrospective study of 51 patients by Lipke *et al.* discovered that predominantly exophytic tumors (>67%) on imaging were more likely to be both papillary RCC and demonstrate a lower Fuhrman grade. These were all pathologically proven malignant tumors in this study, but perhaps this is useful information in a patient with an exophytic tumor desiring active surveillance. Others have looked at potential radiographic predictors of tumor growth. Most studies find no correlation with initial tumor size and growth rate. Multifocal disease and cystic appearance on imaging have also failed to demonstrate any statistical difference with regard to tumor growth rate.[13]

The review by Chawla *et al.* expanded the findings of Rendon *et al.,* which noted the slow growth rate of SRMs and small risk of metastatic potential. In Rendon's small prospective study, they followed up 13 patients for a median of 42 months. Of them, 5 underwent surgical intervention and no patient went on to develop metastatic disease. They concluded that most SRMs grow slowly and metastases are unlikely to arise before the mass shows rapid growth. They suggest that it may be appropriate to explore the hypothesis that increasing the nephrectomy rate for the asymptomatic, incidentally detected, SRM will not reduce mortality rate because the small tumors destined to metastasize do so early. This may deserve some merit because despite earlier diagnosis and treatment of renal masses there has not been a significant increase in cancer specific survival or overall survival.[19]

Additionally, Kouba *et al.* followed up 46 renal masses for a mean of 36 months. A total of 13 patients underwent surgical extirpation. Neither the 13 in the intervention arm nor the remaining patients in the non-intervention arm went on to develop metastatic disease at the 3-year follow-up mark. They reported a correlation between SRM growth rate and age with patients under 60 years of age showing an increased growth rate compared to patients aged over 60 (0.9 vs. 0.6 cm/yr). This observation would support earlier intervention in younger patients. Yet another interesting finding by this group is that of growth rate with relation to symptoms. The mean growth rate of the SRM in the symptomatic group was 1.21 cm/yr compared with 0.52 cm/yr in the asymptomatic group although this did not reach statistical significance (*P* = 0.267). With this information, they suggest that mode of detection may be a prognostic factor independent of stage and grade. Unfortunately, with regard to the SRM, this is not extremely helpful as the overwhelming majority of these tumors are discovered incidentally.

Urologists are being faced with the ever more prevalent question of what to do with the SRM. The gold standard remains surgical excision. The understanding of the natural history of SRMs has suffered secondary to this aggressive approach. With regard to observation, both the clinician and the patient must realize the calculated risk.[16] Until we have a better understanding of tumor biology and growth potential, this option should be reserved for elderly patients with associated significant medical comorbidities. Recent reports in the literature continue to increase the knowledge base for SRMs. Despite these advances, there are no current clinical predictors to identify tumor growth or disease progression.[13] Several studies have looked at age, gender, size at presentation, growth rate, radiographic tumor characteristics, etc.[6–12] Perhaps, the most promising predictors will rely on molecular markers and gene expression. Different types of gene expression patterns have been shown to predict survival.[20,21] However, variation and lack of consistency in studies leave gene expression with limited clinical value at the moment.

Many of these small tumors have been shown to have slow growth rates and low metastatic potential. Hence, an initial time period of surveillance with close follow-up seems quite appealing. This is especially true for the unfit elderly patient. Unfortunately, no discrete cut-off for proceeding to treatment exists in the literature. Current hypothesis being tested involve masses that reach 4 cm in maximum dimension or rapid growth in 1 year.[22] There should be a strict surveillance protocol in place once the decision of observation has been made. We would advocate imaging every 3-6 months with a consistent imaging modality. This eliminates comparing one modality measurements to another.

Yet another option for the SRM is the emerging ablative technology. The most commonly used modalities have been cryoablation and radiofrequency ablation. A recent meta-analysis comparing these two technologies found them to be viable strategies for the SRM.[23] Hegarty *et al.* compared the short-term efficacy and outcomes of the two modalities.[24] At a short median follow-up of 1 year, they found the cancer-specific survival was 98% after cryoablation and 100% after RFA. A further intense review of this technology is beyond the scope of this article. High-intensity focused ultrasound (HIFU) has also been reported for treatment of SRMs; however, at this point it is experimental.

## PERCUTANEOUS BIOPSY

Conventionally, the role of percutaneous biopsy has been controversial with regard to management of renal masses. Recent data looking specifically at SRMs reveal that the percentage of benign findings approaches 30%.[25–27] For this reason, some authors suggest reconsidering the role of biopsy in the management of SRMs.[28] The question surrounding that of renal mass biopsy is that of accuracy. Several new reports suggest that biopsy is quite accurate.[29–32] As noted in Table 2, the sensitivity of detecting malignancy in contemporary biopsy reports is in the 90th percentile. Mode of imaging guidance used does not significantly improve diagnostic yield of biopsies.[29,32] FNA usually yields a lower diagnostic rate (61%) when compared to that of core biopsy (84%).[29] Furthermore, the accuracy of Fuhrman grading based on FNA is significantly insufficient compared with core biopsy (28% vs. 76%, respectively).[33] Several reports found that as tumor size increased so did the diagnostic accuracy of biopsy. This would seem not to favor biopsy of SRMs. The complication rate in recent series is extremely low. Most series report minor complications such as small perinephric hematomas and tiny pneumothoraces managed conservatively.[29,32,33] Devastating complications such as tumor seeding along the needle tract have been estimated to have an overall risk of 0.01% with more recent series reporting no cases of tumor seeding.[34]

**Table 2:** Compilation of recent percutaneous biopsy series

	No. biopsies	Median tumor size (cm)	Complication rate (%)	% Malignant	Sensitivity for malignancy (%)
Volpe *et al.* (2008)	100	2.4	3	79	98
Maturen *et al.* (2007)	152	4.1	1.9	56	97
Schmidbauer *et al.* (2008)	78	3.9	1.3	78	95
Rybicki *et al.* (2003)	115	Not reported*	Not reported	84	90
Thuillier *et al.* (2008)	53	2.57	Not reported	60	96

* Results broken down by range of tumor size, 1-3, 4-6 and >6cm

As reported by Schmidbauer *et al.* and also by Volpe *et al.* in a recent review article, the average sensitivity of FNA for diagnosing malignancy is lower than reports of core biopsies. However, there is controversy with this statement as well. Niceforo *et al.* reported on 23 patients where FNA generated 87% diagnostic accuracy and 100% specificity. Another pertinent issue surrounding percutaneous biopsy is the issue of false-negative results. False-negative rates seem to increase for small renal tumors with Rybicki *et al.,* demonstrating a 13% false-negative rate for tumors between 1 and 3 cm. This is an obvious concern with regard to observation of the SRM. The 1-3 cm range is the exact category in which most of the SRM patients fall. Biopsies of complex cystic lesions also provide diagnostic challenges. As noted previously by Volpe, the diagnostic accuracy can decrease with cystic lesions. Richter found that the combination of FNA and core biopsy was able to histologically identify 90% of 227 Bosniak II-III lesions.

As described, there have been great advances in the area of percutaneous biopsy. The accuracy in recent reports range from 85%-100%.[33,35,37] Core biopsy seems to provide the most accurate reports as demonstrated by Maturen and colleagues, showing a sensitivity for malignancy to be 97%. Fine-needle aspiration has its pitfalls with insufficient samples and nondiagnostic reports. Potential complications of biopsy are tumor seeding, bleeding, arteriovenous fistula, infection and pneumothorax. Contemporary reports demonstrate that minor complications are less than 5% and catastrophic complications are exceedingly rare.[38] This is primarily due to advances in imaging and using coaxial sheaths for multiple passing attempts.[34] A continued drawback of biopsy remains the false-negative result. This is particularly worrisome with SRMs (<3cm). Lechevallier *et al.* demonstrated a biopsy failure rate of 37% with tumors <3 cm vs. 9% with tumors >3 cm. This is supported by Rybicki *et al.* who found a 13% false-negative rate among tumors ranging from 1 to 3 cm compared with only a 2.4% false-negative rate among tumors between 4 and 6 cm. With the current data, the role of biopsy in managing small renal tumors has yet to be determined. As molecular markers, gene expression, and translational research improves, we hope that its role will become more precise. An ideal future scenario would involve biopsy of a mass, identify its tumor biology and then match the treatment modality accordingly.

## CONCLUSION

With the metastatic potential of RCC, select elderly surgically unfit patients can be considered for watchful waiting. The risk of metastasis during watchful waiting for SRMs is only about 1%. If watchful waiting is planned, 3-6 monthly imaging to monitor size and tumor characteristics is recommended. The natural history and metastatic potential of the SRM will become more evident as more large prospective trials are produced. Percutaneous image-guided biopsy of renal masses can be performed safely with a greater than 90% sensitivity to detect malignancy. However, its role in the management of SRMs continues to evolve.

Small renal masses in younger and healthy patients could undergo image-guided needle biopsy to confirm the pathology before definitive management. It is our practice that such patients with small renal malignancy are advised to undergo nephron-sparing surgery. Renal tumor ablation, i.e., cryoablation or radiofrequency ablation are also options, though we would prefer ablation in the older patient. Watchful waiting in healthy young patients with proven renal cancer is certainly not the standard of care. Though the risk of metastasis is very minimal with watchful waiting, the tumors must be followed up with imaging periodically to confirm that there is no significant tumor growth. The costs involved with imaging and the possible radiation risk with several CTs must be considered.
